# Comparative efficacy and safety of paricalcitol versus vitamin D receptor activators for dialysis patients with secondary hyperparathyroidism: a meta-analysis of randomized controlled trials

**DOI:** 10.1186/s12882-017-0691-6

**Published:** 2017-08-25

**Authors:** Yifeng Xie, Peiling Su, Yifan Sun, Hongsheng Zhang, Rong Zhao, Liang Li, Lanfen Meng

**Affiliations:** 1 Department of Nephrology, The Third Affiliated Hospital of Guangxi University of Chinese medicine, No.32, Jie-Fang-Bei Road, Cheng-Zhong District, Liuzhou, Guangxi 545001 People’s Republic of China; 2 Department of Clinical Laboratory, The Third Affiliated Hospital of Guangxi University of Chinese medicine, Liuzhou, Guangxi People’s Republic of China; 3 Department of Orthopaedics, The Third Affiliated Hospital of Guangxi University of Chinese medicine, Liuzhou, Guangxi People’s Republic of China

**Keywords:** Paricalcitol, Vitamin D receptor activator, Secondary hyperparathyroidism (SHPT), Hemodialysis, Peritoneal dialysis

## Abstract

**Background:**

Secondary hyperparathyroidism (SHPT) is a severe complication for dialysis patients. Vitamin D receptor activators (VDRAs) are used to treat SHPT, but the comparative efficacy and safety between paricalcitol and other vitamin D receptor activators for management of SHPT in dialysis patients has been unproven.

**Methods:**

We searched PubMed, Embase, and the Cochrane Library for the time period through June 2017 to identify randomized controlled trials that evaluated paricalcitol compared with other VDRAs for treatment of SHPT. The primary outcome was the percentage of patients with target reduction of intact parathyroid hormone (iPTH) from baseline. Secondary outcomes included incidences of hypercalcemia and hyperphosphatemia. The random-effects model was used to estimate relative risks (RRs) with 95% confidence intervals (CIs).

**Results:**

Eight studies (*N* = 759) were eligible for final inclusion. Compared with other VDRAs, no significant differences were found in the percentage of patients with target reduction of intact parathyroid hormone (iPTH) from baseline for paricalcitol treatment of SHPT in dialysis patients (RR, 1.01; 95% CI, 0. 87–1.18; *p* = 0.85). There were no differences in the incidence of hypercalcemia (RR, 0.95; 95% CI, 0.74–1.21; *p* = 0. 65) and hyperphosphatemia (RR, 0.94; 95% CI, 0.77–1.16; *p* = 0.58).

**Conclusions:**

The presently available evidence is insufficient to draw a conclusion regarding whether paricalcitol therapy has a comparative efficacy and safety over other VDRAs for treating dialysis patients with SHPT. Large-sample, well-conducted, high-quality RCTs with patient-level outcomes (i.e., mortality) are urgently needed.

**Electronic supplementary material:**

The online version of this article (10.1186/s12882-017-0691-6) contains supplementary material, which is available to authorized users.

## Background

Secondary hyperparathyroidism (SHPT) is one of the most common abnormalities of the chronic kidney disease-mineral and bone disorders (CKD-MBD) syndrome. Abnormalities in serum calcium, phosphorus, intact PTH, and vitamin D deficiency are common in dialysis patients with SHPT [1]. Increased levels of serum calcium, phosphorus, and intact PTH had been associated with increased all-cause and cardiovascular mortality [2–6]. However, the DOPPS study indicated that there was a lower mortality risk for calcium, phosphorus, and intact PTH within specific ranges [7]. Based on the epidemiological and clinical evidences, Kidney Disease: Improving Global Outcomes (KDIGO) recommended a target range of serum calcium, phosphorus, and intact PTH for treating dialysis patients with SHPT [1].

To achieve current targets for the key mineral parameters set by KDIGO guidelines, a combination of dietary phosphorus restriction, phosphate binders, vitamin D receptor activators (VDRAs), and adequate dialysis was adopted. Among the VDRAs used for management of SHPT in dialysis patients, paricalcitol is the most potential vitamin D analog. The observational study by Teng et al. indicated a significant survival advantage of paricalcitol over calcitriol for long-term hemodialysis patients [8]. However, whether or not paricalcitol has a comparative efficacy and safety with other VDRAs for treating SHPT in dialysis patients, based on RCTs, is unknown.

Previous meta-analyses focused mainly on comparisons between paricalcitol and placebo [9–11] in both dialysis patients [12] and patients not requiring dialysis [9, 10, 13]. We performed a meta-analysis making a head-to-head comparison to check therapeutic advantages of paricalcitol over other VDRAs in dialysis patients with SHPT.

## Methods

We conducted a meta-analysis of randomized controlled trials in compliance with the PRISMA (Preferred Reporting Items for Systematic Reviews and Meta-Analyses statement) guidelines [14]. The present meta-analysis had no registered protocol.

### Data sources and Searches

Literature searches for randomized, controlled trials of vitamin D receptor activators in CKD 5D (dialysis) patients with secondary hyperparathyroidism were performed in PubMed, Embase, and the Cochrane Library for the period through January 2016. The Medical Subject Headings (MeSH) terms and corresponding keywords were used for the electronic searches. The search terms used were (MeSH exp. “Kidney Disease,” “Kidney Failure,” “Chronic Kidney Failure,” and the keywords “chronic kidney”, “chronic renal”, “dialysis” and “hyperparathyroidism”), and (MeSH exp. “Vitamin D” and keywords “vitamin D” and “paricalcitol”). The complete search strategy is outlined in Additional file 1. The cited references of published primary studies and reviews were manually screened to identify relevant trials. To ensure literature saturation, we reran the searches on June 25, 2017. The ClinicalTrials.gov registry (https://clinicaltrials.gov/) was also searched. Trials were identified without language restriction.

### Study selection

Two authors (Yifeng Xie and Peiling Su) independently conducted the search, screened the titles and abstracts, and checked full text for eligibility. Discrepancies between authors were resolved by consensus. Published RCTs meeting the following inclusion criteria were included: (1) population: chronic kidney disease 5D adult patients (hemodialysis or peritoneal dialysis) with secondary hyperparathyroidism; (2) intervention: paricalcitol therapy; (3) comparison: vitamin D analogues such as calcitriol, alfacalcidol, maxacalcitol, et al.; and (4) one or more the following outcomes: percentage of participants with target reduction in intact parathyroid hormone (iPTH) from baseline; incidence of hypercalcemia, hyperphosphatemia, and elevation in calcium phosphorus product; all-cause mortality and end-of-treatment serum phosphorus, calcium, and iPTH levels. Studies that did not meet inclusion criteria (i.e., participants were pediatric patients or comparison was placebo.) were excluded.

### Data extraction and Quality assessment

Data extraction was performed by Yifeng Xie and Peiling Su using a standardized data extraction sheet (Office Excel®, Microsoft® Corporation) and confirmed independently by another author (Hongsheng Zhang). Three authors double-checked data entry independently. Any disagreements between authors were resolved by discussion and consensus. The collected data included the following: first author, year of publication, country, number of patients, follow-up, age, baseline mineral and bone disorders, laboratory values (serum calcium, phosphorus, and iPTH), types of phosphate binders used, dosing schedule, routine of administration, risk of bias data, and outcomes data. Risk of bias data were those that described randomization, double-blinding, and dropouts and withdrawals. Predefined primary outcome was the percentage of patients with target reduction in iPTH from baseline. Secondary outcomes included incidences of hypercalcemia, hyperphosphatemia, and elevation in the calcium-phosphorus product; end-of-treatment serum iPTH, phosphorus, and calcium; and all-cause mortality. For crossover trials, the data from the first period were used [15].

Two authors (Yifeng Xie and Peiling Su) independently assessed the risk of bias of the included studies using a validated Jadad 5-point scale [16, 17]. The scale consists of three items describing randomization (0–2 points), masking (0–2 points), and dropouts and withdrawals (0–1 points) in the report of a randomize controlled trial. The scale ranged from 0 to 5, with higher scores showing better reporting. The trials were rated high quality when the score was higher than 2, or low quality when the score was 2 or below, out of a maximum score of 5.

### Statistical analysis

We summarized effect estimates as relative risks (RRs) for dichotomous outcomes and weighted mean differences for continuous outcomes with 95% CIs. We assessed heterogeneity of treatment effects across studies with the I^2^ statistic and I^2^ > 50% significant heterogeneity [18]. We pooled effect estimates using a random-effects model, taking clinical heterogeneity between studies into account. To examine the influence of various factors on the treatment effects of paricalcitol therapy for dialysis patients with secondary hyperparathyroidism, we performed post-hoc subgroup analyses according to baseline serum PTH levels (PTH > 68.4 pmol/L versus PTH < 68.4 pmol/L, with the cut-off value set as 9 times the upper normal limit for the assay), types of dialysis (hemodialysis versus peritoneal dialysis), routine of administration (oral versus intravenous), sample size (*n* > 100 for large sample studies versus *n* < 100 for small sample studies), and risk of bias (low quality versus high quality). Publication bias was evaluated visually by funnel plot and quantitatively by Egger’s test and Begg’s test [19, 20]. We performed all statistical analyses using RevMan 5.3 (Nordic Cochrane Centre). *P* < .05 was considered statistically significant.

## Results

### Trial selection

**Fig. 1:**
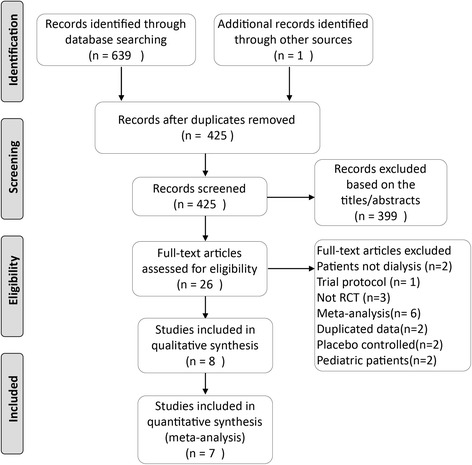
PRISMA flow diagram for study selection

The PRISMA statement flowchart shows the process of study identification, screening, selection, and reasons for exclusion, as shown in Fig. 1. The combined search identified a total of 640 records, 614 of which were excluded after removing duplicates and screening the titles and abstracts. Full-text assessment of 26 potentially eligible articles identified 8 studies. Finally, 8 RCTs [21–28] were included in the meta-analysis.

### Trials characteristics

Characteristics of patients and interventions of the included trials are summarized in Table 1. The sample size ranged from 18 to 263, with a total of 759 patients. Only two trials [21, 27] had more than 100 participants. Of the included trials, seven compared paricalcitol therapy with non-selective vitamin D receptor activators, including calcitriol and alfacalcidol, and the remaining one [21] compared paricalcitol therapy with selective vitamin D receptor activator (maxacalcitol).

**Table 1:** Charateristics of included studies in the meta-analysis

Study, year	Subjects, N(p/c)	Follow-up(weeks)	Mean age (years)	Type of Dialysis	Baseline MBD Labs*	Phosphate binders	Dosing schedule		Routine of administration	Outcomes
							Paricalcitol	Control		
Sprague 2003 [27]	263(130/133)	32	56.7	HD	Ca 2.25 (2.25) mmol/LP 1.91 (1.87) mmol/LPTH 68.7 (71.6) pmol/L	calcium carbonate calcium acetate	Initial dose 0.04 μg/kg and increment of 0.04 μg/kg every 4 weeks. Maximum dose was 0.24 μg.	Initial dose 0.01 μg/kg and increment of 0.01 μg/kg every 4 weeks. Maximum dose was 0.06 μg.	IV	1,2,3,4
Gafor 2009 [26]	25(13/12)	12	48.2	HD	Ca 2.29 (2.30) mmol/LP 1.58 (1.65) mmol/LPTH 136.8 (128.1) pmol/L	calcium-containing	Initial dose 0.04 μg/kg and increment of 0.04 μg/kg every 3 weeks.	Initial dose 0.01 μg/kg and increment of 0.01 μg/kg every 3 weeks.	IV	5,6,7
Lund 2010 [25]	18(9/9)	5	51.1	HD	Ca 2.17 (2.22) mmol/LP 1.58 (1.71) mmol/LPTH 17.2 (14.6) pmol/L	Sevelamer HCL	Initial dose 0.06 μg/kg	Initial dose 0.02 μg/kg	IV	6,7
Hansen 2011 [24]	86(45/41)	16	63.7	HD	Ca 1.15 (1.16) mmol/L^a^P 1.46 (1.48) mmol/LPTH 57.0 (60.0) pmol/L	calcium-containing sevelamer Lanthanum	Initial dose 9 μg/week, The median (range) final doses for the last 4 weeks: 18.1 μg/week (6.8–60.0 μg/week)	Initial dose 3 μg/week, The median (range) final doses for the last 4 weeks: 5.3 μg/week (0.0–33.0 μg/week)	IV	1,2,3,4,5,6,7,8
Ong 2013 [23]	66(36/30)	24	46.3	HD	Ca 2.17 (2.12) mmol/LP 1.87 (1.72) mmol/LPTH 52.5 (59.2) pmol/L	calcium carbonate	Initial dose:(iPTH/12.7)μg, The mean maximal dose 29.6 μg; The mean weekly dose 20.9 μg	Initial dose:(iPTH/38.1)μg, The mean maximal dose 9.9 μg; The mean weekly dose 7.1 μg	PO	1,2,4,6,7
Jamaluddin 2014 [22]	26(12/14)	15	48.3	PD	Ca 2.24 (2.25) mmol/LP 1.65 (2.02) mmol/LPTH 85.7 (98.9) pmol/L	calcium carbonate	Initial dose = iPTH/7 every other day up to a maximum initial dose of 32 μg.	Initial dose 5 μg daily.	PO	1,2
Akizawa 2015 [21]	255(127/128)	12	61.5	HD	Ca 2.28 (2.28) mmol/LP 1.68 (1.69) mmol/LPTH 55.5 (55.9) pmol/L	calcium-containing;non-Ca containing	Initial dose 2 μg, adjusted ±1 μg based on protocol-specified criteria up to a maximum of 7 μg.	Initial dose 5 μg (iPTH < 53 pmol/L) or 10 μg(iPTH > 53 pmol/L) adjusted ±2.5 μg based on protocol-specified criteria up to a maximum of 20 μg.	IV	1,2,3
Veceric 2016 [28]	20(10/10)	12	56	HD	Ca 2.23 (2.04) mmol/L P 1.56 (1.54) mmol/L PTH 63.8 (66.0) pmol/L	non-Ca containing	Initial dose 2 μg; three times per week.	Initial dose 0.5 μg; three times per week.	PO	NR

*P* paricalcitol, *C* control; The control was calcitriol or alfacalcidol or maxacalcitol. *NR* not reported. *MBD* mineral and bone disease. *PO* oral, *IV* intravenous, *HD* hemodialysis, *PD* peritoneal dialysis, *iPTH* intact parathyroid hormone.

Symbols: *Paricalcitol(control); a: Ionized calcium

Outcomes: 1. Target reduction of iPTH from baseline; 2. Hypercalcemia; 3. Hyperphosphatemia; 4. Elevation in the calcium-phosphorus product; 5. End-of-treatment serum PTH; 6. End-of-treatment serum phosphorus; 7. End-of-treatment serum calcium; 8. All-cause mortality

Five trials [21–24, 27] reported the primary outcome, which was the percentage of patients with target reduction in intact parathyroid hormone (iPTH) from baseline. Of the five trials reporting the primary outcome, two trials [23, 24] used a > 30% reduction as target reduction, whereas the remaining three trials [21, 22, 27] used a > 50% reduction. All included trials used different cut-off values to define hypercalcemia, ranging from 2.63 mmol/L (10.5 mg/dL) to 2.90 mmol/L (11.5 mg/dL). Five trials [21–24, 27] reported incidence of hypercalcemia, one [24] of them using serum ionized calcium and the others serum total calcium. Only one trial [24] presented all-cause mortality data and others did not report patient-level outcomes (i.e., all-cause mortality, cardiovascular mortality, and fractures). One trial [28] did not present any outcomes, either primary or secondary. All RCTs had a short duration of follow-up, lasting from 5 to 32 weeks.

### Risk of bias assessment

The details of risk of bias are listed in Table 2. Overall, four trials [22, 23, 26, 28] were categorized as low methodological quality and four [21, 24, 25, 27] as high methodological quality. Only one trial [24] described appropriate allocation concealment. Three trials [21, 25, 27] reported double-blinding, and all trials except one [28] reported drop-outs and withdrawals.

**Table 2:** Assessing risk of bias of the included studies by validated Jadad 5-point scale

Author	Publication year	Country	Sample size (n)	Study Design	Randomization (0–2 points)	Double-blind (0–2 points)	Drop-outs and withdrawals (0–1 points)	Jadad score
Sprague [27]	2003	USA	263	multicenter, double-blind, parallel	1	2	1	4
Gafor [26]	2009	Malaysia	25	single-center, open-labelled, parallel	1	0	1	2
Lund [25]	2010	USA	18	single-center, double-blind, crossover	1	2	1	4
Hansen [24]	2011	Denmark	86	multicenter, open-labelled, crossover	2	0	1	3
Ong [23]	2013	Malaysia	66	multicentre, open-labelled, parallel	1	0	1	2
Jamaluddin [22]	2014	Malaysia	26	single-center, open-labelled, parallel	1	0	1	2
Akizawa [21]	2015	Japan	255	multicenter, double-blind, parallel	1	2	1	4
Veceric [28]	2016	Slovenia	20	single-center, open-labelled, parallel	1	0	0	1

### Paricalcitol treatment versus vitamin D receptor activator treatment

#### Primary outcome

**Fig. 2:**
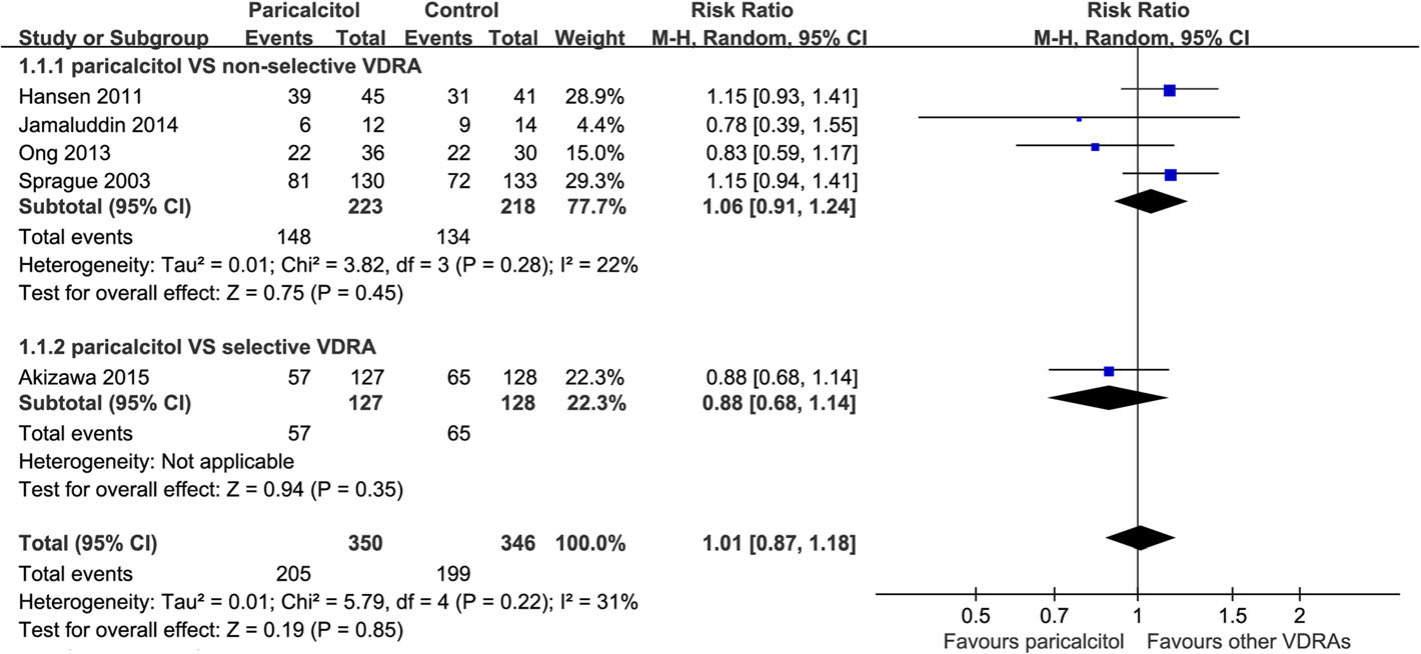
Effect of paricalcitol on suppressing iPTH as compared to other VDRAs

Five trials [21–24, 27] with a total of 696 patients were included for the outcome of the percentage of patients with target reduction in iPTH from baseline. Compared to either non-selective vitamin D receptor activators (calcitriol and alfacalcidol) or selective vitamin D receptor activator (maxacalcitol), paricalcitol had similar efficacy in suppressing the iPTH of dialysis patients with SHPT (Fig. 2.). The RR was 1.01 (95% CI: 0.87–1.18; *p* = 0.85). The statistic I^2^ was 31%, indicating low heterogeneity.

There was no significant difference in suppressing iPTH with paricalcitol in comparison to other VDRAs for all subgroup analyses. Details of subgroup analyses were presented in Table 3.

**Table 3:** Subgroup analyses of paricalcitol compared with other VDRAs for target reduction of iPTH from baseline

Subgroup	No.patients	No.trials	Relative Risk(95% CI)	*P* Value	I^2^(%)	Test of Interaction, P
Total	696	5	1.01 (0.87,1.18)	0.85	31	Not applicable
Baseline iPTH level						
< 68.4 pmol/L	407	3	0.97 (0.78,1.20)	0.77	51	0.49
> 68.4 pmol/L	289	2	1.09 (0.84,1.42)	0.51	13	
Types of dialysis						
Hemodialysis	670	4	1.03 (0.87,1.20)	0.76	41	0.44
Peritoneal dialysis	26	1	0.78 (0.39,1.55)	0.47	31	
Routine of administration						
Oral	92	2	0.82 (0.61,1.11)	0.21	0	0.14
Intravenous	604	3	1.07 (0.91,1.26)	0.42	38	
Sample size						
Small, *n* < 100	178	3	0.98 (0.75,1.28)	0.87	42	0.82
Large, *n* > 100	518	2	1.02 (0.79,1.32)	0.88	60	
Risk of bias						
Low quality	92	2	0.82 (0.61,1.11)	0.21	0	0.14
High quality	604	3	1.07 (0.91,1.26)	0.42	38	

#### Secondary outcomes

**Fig. 3:**
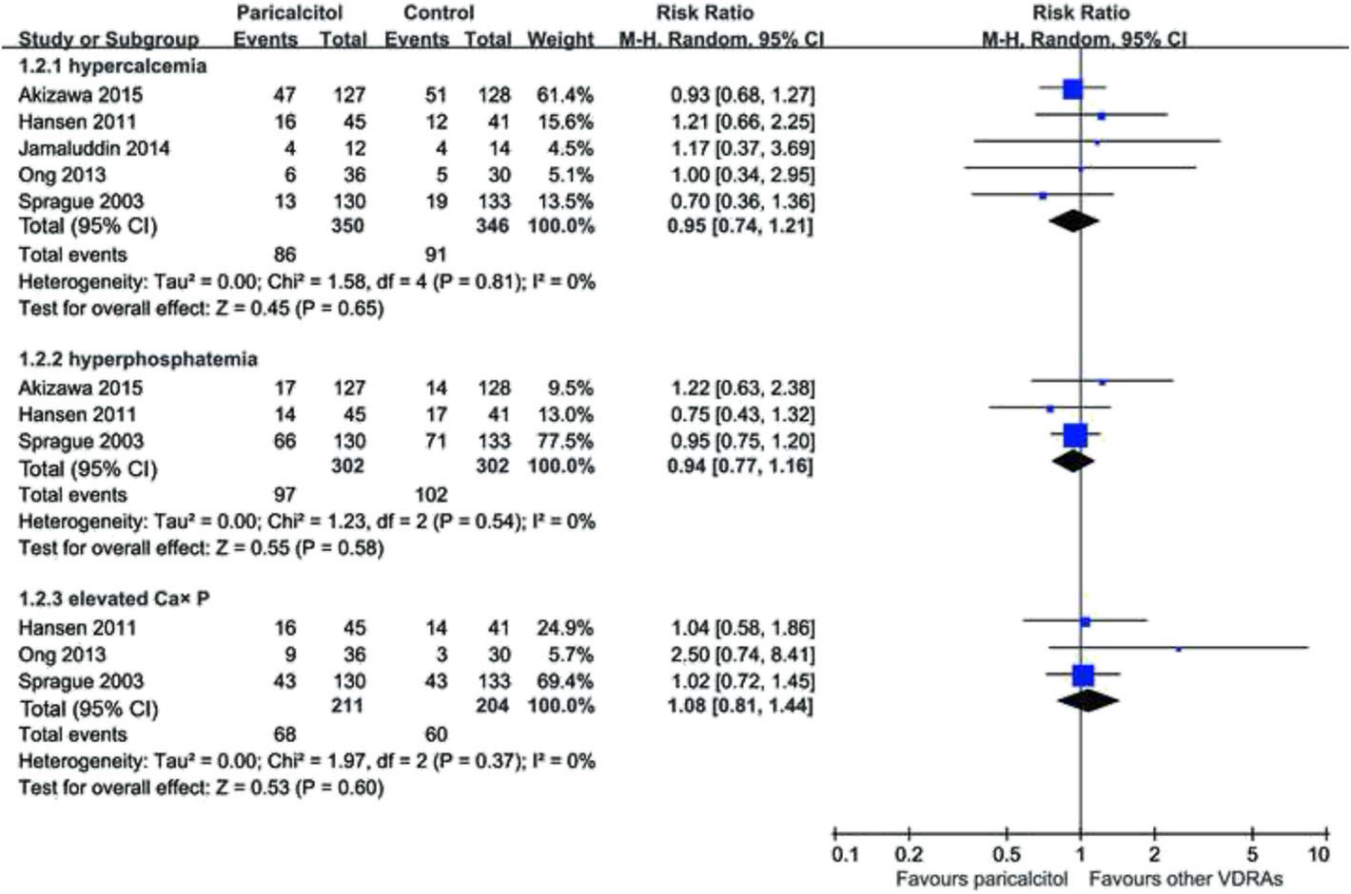
Risk of hypercalcemia, hyperphosphatemia, and elevation in the calcium-phosphorus product with paricalcitol comparing to other VDRAs

There was no significant difference in the risk of hypercalcemia, hyperphosphatemia, and elevation in the calcium-phosphorus product with paricalcitol in comparison to other VDRAs, as shown in Fig. 3. The pooled RRs with 95% CI for incidence of hypercalcemia, hyperphosphatemia, and elevation in the calcium-phosphorus product were 0.95 (0.74–1.21; *p* = 0.65), 0.94 (0.77–1.16; *p* = 0.58), 1.08 (0.81–1.44; *p* = 0.60), respectively. The statistic I^2^ was 0% for all three outcomes.

**Fig. 4:**
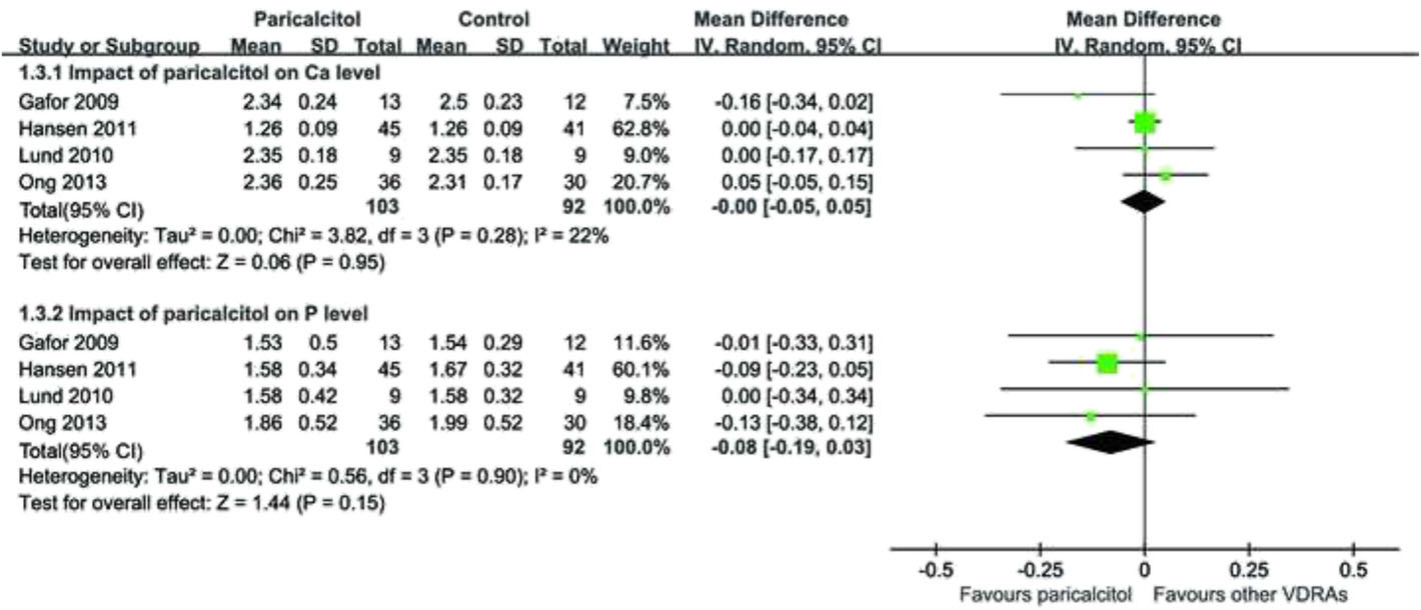
Effect of paricalcitol on end-of-treatment serum calcium and phosphorus as compared to other VDRAs

There was no significant difference in end-of-treatment serum calcium and phosphorus with paricalcitol in comparison to other VDRAs, as shown in Fig. 4. The pooled mean differences (MDs) with 95% CI were 0 (−0.06–0.05; *p* = 0.95) and −0.08 (−0.19–0.03; *p* = 0.15), respectively. The statistics I^2^ were 24% and 0%, respectively.

## Discussion

This meta-analysis demonstrates that there was no significant difference in effectiveness and safety of paricalcitol therapy in comparison to other VDRAs based on the biochemical end points when treating adult dialysis patients with secondary hyperparathyroidism. Comparisons based on patient-centered outcomes are not possible presently because of the lack of RCTs providing data on patient-level outcomes, such as mortality, cardiovascular death, fracture, and quality of life.

KDIGO’s recommendations on dialysis patients with SHPT are to lower elevated phosphorus levels toward the normal range while maintaining intact PTH levels in the range of ~2–9 times the upper normal limit for the assay and maintaining serum calcium in the normal range [1]. To reach the treatment target, limited dietary intake of phosphorus, phosphate binder, Vitamin D compounds, and adequate dialysis are adopted. A meta-analysis of 14 observational studies demonstrated that vitamin D compound therapies reduced mortality in CKD patients, particularly in those suffering from secondary hyperparathyroidism [29]. A study by Teng et al. showed that paricalcitol had a survival advantage over calcitriol in patients undergoing long-term hemodialysis [8]. However, all the included trials in this meta-analysis did not report patient-level outcomes. Comparisons based on the biochemical end points did not demonstrate an advantage of paricalcitol therapy over other VDRAs therapies. Several factors may affect both direction and size of the effect estimates and account for a risk of bias across the included studies. First, there was a diverse spectrum of iPTH at the baseline, ranging from 14.6 pmol/L to 136.8 pmol/L. Second, the majority of the included studies had a small sample size (*n* < 100) with inadequate power. Third, there were two target-reduction values of iPTH (>30% and >50%) for the definition of primary outcome. Also, there were different cut-off values for the definition of hypercalcemia. Fourth, different types of phosphate binders were used in the included trials. A meta-analysis showed that non-calcium-containing phosphate binders had an advantage of a decrease in hypercalcemia in dialysis patients over the calcium-containing phosphate binders [30]. Fifth, dose ratios of 4:1 and 3:1, or an initial dose, depending on iPTH level, were used. Thus, the potential dose-response might affect the effect size [31]. Sixth, durations of follow-up were too short for the included studies. Seventh, fibroblast growth factor 23 (FGF 23) plays an important role in the mineral and bone disorders of CKD patients [32–35]. However, the included studies of this meta-analysis had no information on the serum FGF23 levels of patients enrolled in the studies, except that one study provided incomplete data of FGF23 [36]. Any significant within-group or between-group differences of FGF23 may affect the treatment effects of the included trials.

The negative result of our meta-analysis is consistent with previous meta-analyses [12, 37, 38]; however, this meta-analysis reveals several differences. First, the population is limited to CKD adult dialysis patients with SHPT. Patients not on dialysis were excluded because of significant differences of the association between serum phosphorus/calcium/iPTH and mortality in these two types of patients [1, 39, 40]. Second, active VDRA treatments were chosen as controls in this meta-analysis; thus, a direct comparison between paricalcitol and other VDRAs is made to check the superiority. Third, compared with recently published meta-analyses [37], this meta-analysis adds a comparison between selective vitamin D analogues (paricalcitol versus maxacalcitol).

This meta-analysis has the following limitations: First, the number of included studies is small and the studies had small sample sizes and short follow-up lengths. Second, the number of included trials is too small to evaluate publication bias of our meta-analysis, either visually by funnel plot or quantitatively by Egger’s test [20] and Begg’s test [19]. There may be potential publication bias in this meta-analysis. Third, comparisons between paricalcitol and other VDRAs are based on biochemical end points. Patient-level outcomes are not available in the included studies. Fourth, since the control included both non-selectvie VDRAs and selective VDRAs, this could introduce some heterogeneity for the summarized effect.

## Conclusions

The evidence that is presently available is insufficient to draw a conclusion regarding whether paricalcitol therapy has a comparative efficacy and safety over other VDRAs for treating dialysis patients with SHPT. Large-sample, well-conducted, high-quality RCTs with patient-level outcomes (i.e., mortality) are urgently needed.

## Additional file

Additional file 1: Search Strategy. (DOCX 18 kb)

## Abbreviations

CI: Confidence interval
CKD: Chronic kidney disease
CKD-MBD: Chronic kidney disease–mineral and bone disorder
iPTH: Intact parathyroid hormone
KDIGO: Kidney Disease: Improving Global Outcomes
MD: Mean difference
MeSH: Medical Subject Headings
PRISMA: Preferred Reporting Items for Systematic Reviews and Meta-Analyses
RCT: Randomized controlled trial
RR: Relative risk
SHPT: Secondary hyperparathyroidism
VDRA: Vitamin D receptor activator
