# Supplementary material for: Comparative efficacy and safety of paricalcitol versus vitamin D receptor activators for dialysis patients with secondary hyperparathyroidism: a meta-analysis of randomized controlled trials
Source: BMC Nephrol. 2017 Aug 25;18:272. doi: 10.1186/s12882-017-0691-6 (PMC5574209; doi:10.1186/s12882-017-0691-6)
Supplement: Additional file 1: — Search Strategy. (DOCX 18 kb) [file 12882_2017_691_MOESM1_ESM.docx]

***Search Strategies***

| PubMed | |
| --- | --- |
| 1 | Kidney Disease[MeSH] |
| 2 | Kidney Failure[MeSH] |
| 3 | Kidney Failure, Chronic[MeSH] |
| 4 | (chronic renal or chronic kidney or end stage renal disease or ESRD or CRF ) |
| 5 | (hemodialysis or haemodialysis or dialysis or peritoneal dialysis or CAPD or CCPD or APD ) |
| 6 | (secondary hyperparathyroidism or hyperparathyroidism) |
| 7 | 1 or 2 or 3 or 4 or 5 or 6 |
| 8 | Vitamin D[MeSH] |
| 9 | (paricalcitol or 19-Nor-1-alpha-25-dihydroxyvitamin D2 or doxercalciferol or calcitriol or maxacalcitol or alfacalcidol or Vitamin D) |
| 10 | 8 or 9 |
| 11 | (randomized controlled trial [pt] OR controlled clinical trial [pt] OR randomized [tiab] OR placebo [tiab] OR clinical trials as topic [mesh: noexp] OR randomly [tiab] OR trial [ti]) NOT (animals [mh] NOT humans [mh]) |
| 12 | 7 AND 10 AND 11 |
| 13 | paricalcitol or 19-Nor-1-alpha-25-dihydroxyvitamin D2 |
| 14 | 12 AND 13 |
|  |  |
|  |  |
| Cochrane Library | |
| 1 | MeSH descriptor Kidney Disease explode all trees |
| 2 | MeSH descriptor Kidney Failure explode all trees |
| 3 | MeSH descriptor Kidney Failure Chronic explode all trees |
| 4 | (chronic renal or chronic kidney or end stage renal disease or ESRD or CRF ) |
| 5 | (hemodialysis or haemodialysis or dialysis or peritoneal dialysis or CAPD or CCPD or APD ) |
| 6 | (secondary hyperparathyroidism or hyperparathyroidism) |
| 7 | 1 or 2 or 3 or 4 or 5 or 6 |
| 8 | MeSH descriptor vitamin D explode all trees |
| 9 | (paricalcitol or 19-Nor-1-alpha-25-dihydroxyvitamin D2 or doxercalciferol or calcitriol or maxacalcitol or alfacalcidol or Vitamin D) |
| 10 | 8 or 9 |
| 11 | 7 AND 10 |
| 12 | paricalcitol or 19-Nor-1-alpha-25-dihydroxyvitamin D2 |
| 13 | 11 AND 12 |
|  |  |
|  |  |
| Embase | |
| 1 | 'Kidney Disease'/exp |
| 2 | 'Kidney Failure'/exp |
| 3 | 'Chronic Kidney Failure'/exp |
| 4 | ('chronic renal' or 'chronic kidney' or 'end stage renal disease' or ESRD or CRF ) |
| 5 | (hemodialysis or haemodialysis or dialysis or 'peritoneal dialysis' or CAPD or CCPD or APD ) |
| 6 | ('secondary hyperparathyroidism' or hyperparathyroidism) |
| 7 | 1 or 2 or 3 or 4 or 5 or 6 |
| 8 | 'Vitamin D'/exp |
| 9 | (paricalcitol or '19-Nor-1-alpha-25-dihydroxyvitamin D2' or doxercalciferol or calcitriol or maxacalcitol or alfacalcidol or Vitamin D) |
| 10 | 8 or 9 |
| 11 | random* OR blind* OR placebo |
| 12 | 7 AND 10 AND 11 |
| 13 | paricalcitol or 19-Nor-1-alpha-25-dihydroxyvitamin D2 or 19-nor-1,25(OH)2D2 |
| 14 | 12 AND 13 |
